# DLR-YOLO: A High-Accuracy Lightweight Object Detector for Complex Underground Coal Mine Environments

**DOI:** 10.3390/s26103119

**Published:** 2026-05-15

**Authors:** Xiaohang Cai, Ruimin Wang, Jianhui Zhang, Junjie Zeng

**Affiliations:** 1 School of Cyberspace Security, Zhengzhou University, Zhengzhou 450002, China; xhcai@gs.zzu.edu.cn (X.C.); zengjj_lab@zzu.edu.cn (J.Z.); 2 School of Computer and Artificial Intelligence, Zhengzhou University, Zhengzhou 450001, China; iermwang@zzu.edu.cn; 3 Songshan Laboratory, Zhengzhou 450002, China

**Keywords:** DLR-YOLO, underground coal mine object detection, lightweight object detector, YOLOv11n, multi-scale feature extraction, feature fusion

## Abstract

Object detection in underground coal mines is plagued by critical challenges, including low illumination, high dust-induced noise, extensive target scale variation, frequent occlusion, and fragmented target feature representation, which commonly result in severe missed detections and insufficient detection confidence. To tackle these bottlenecks, this study proposes DLR-YOLO, a high-performance lightweight object detector built upon the YOLOv11n baseline, with three core optimized modules. Specifically, a dynamic multi-scale global perception enhancement module (DMGPEM) is embedded in the backbone to realize adaptive multi-scale feature extraction under low-light conditions; a lightweight cross-attention (LCA) module is integrated into the neck to achieve efficient fusion of shallow detail features and deep semantic features while suppressing dust-related noise; and a Reparameterized stem (RepStem) module is developed for initial feature extraction to minimize critical information loss during downsampling. Experimental results on our self-collected and annotated in-house underground coal mine dataset demonstrate that DLR-YOLO achieves 94.4% mAP@50 and 66.7% mAP@50–95, corresponding to 3.5 and 5.7 percentage point improvements over the YOLOv11n baseline, respectively. Ablation studies further validate the independent effectiveness of each proposed module. Meanwhile, the detector maintains a lightweight architecture with only 2.7M parameters and 6.6 GFLOPs, and reaches an inference speed of 157.1 FPS, outperforming several state-of-the-art real-time detectors, including YOLOv12, YOLOv13, and RT-DETR, on the same dataset. These findings confirm that DLR-YOLO provides a robust, high-performance technical foundation for real-time safety monitoring systems in complex underground coal mine environments.

## 1. Introduction

Coal remains a fundamental energy source in the global energy structure, playing an irreplaceable role in industrial production and power generation. As the world’s largest coal producer and consumer, China maintains coal’s share in its energy mix at over 56%, with underground mining operations consistently ranking among the highest globally [1,2]. With the continuous advancement of the “Intelligent Mine” strategy, underground coal mine operations are rapidly evolving toward automation and intelligence, where vision-based safety monitoring systems have become a core supporting component of smart mine construction [3]. As a fundamental task in computer vision, object detection is the core enabling technology for intelligent mine safety monitoring systems. Its performance directly affects the reliability of downstream applications, including underground personnel identification, real-time equipment status monitoring, and safety hazard early warning, thus providing key technical support for the safe development of intelligent coal mining [4,5,6].

Object detection technology has evolved through three key stages, which lay the technical foundation for our underground coal mine safety monitoring research. The first stage is traditional handcrafted feature-based detection methods, represented by the HOG+SVM framework [7]; such methods rely on manually designed features, with detection performance degrading significantly in the low-light and high-noise conditions common in underground coal mines [8]. The second stage is deep learning-based two-stage detectors, represented by the R-CNN series [9]; while Faster R-CNN optimizes detection speed via the RPN structure [10], its heavy computational overhead cannot meet the real-time deployment requirements of underground edge devices. The third stage is single-stage detectors, represented by YOLO [11] and SSD [12]; the end-to-end YOLO framework [13] achieves an excellent balance between accuracy and speed, making it the most suitable baseline for our underground detection scenario.

Underground coal mine environments are highly complex and pose unique challenges for object detection. Low illumination creates minimal grayscale contrast between targets and the background, blurring feature edges and weakening feature discriminability [14], while high dust concentrations introduce severe image noise and color deviation that corrupt target feature integrity [15]. Dense equipment layout and dynamic personnel movement in confined tunnels cause frequent target occlusion and incomplete feature representation, and the large-scale variation across common detection targets (e.g., miners, helmets, towlines) further elevates detection difficulty. These combined factors lead to high missed detection rates, false positives, and low detection confidence in existing detectors, especially in texture-scarce, high-noise scenes. Current methods struggle to simultaneously meet the three core requirements for underground edge deployment—real-time inference, high robustness to environmental interference, and a lightweight model architecture—limiting performance improvements for intelligent coal mine safety monitoring systems.

In recent years, numerous researchers have conducted improvement studies on object detection algorithms tailored to the specific characteristics of underground coal mine environments. Some studies have introduced attention mechanisms to enhance the model’s focus on key features. For instance, Fu et al. [16] proposed YOLOv8n-FADS integrating the TripletAttention mechanism, improving helmet detection accuracy, though missed detections persist under heavy dust and multi-target occlusion scenarios. Fan et al. [17] proposed CM-YOLOv8, achieving a lightweight design through L1 norm pruning, but with limited capability for capturing weak features. Zhang et al. [18] proposed GCB-YOLOv11, optimizing feature extraction and fusion through the GSConv and BiFPN modules, though its parameter compression and robustness to noise, dust, occlusion, and uneven illumination still have room for improvement. Sun et al. [19] optimized dust-interfered image quality through ACE dehazing preprocessing, integrating the DCNv deformable convolution and SimAM parameter-free attention mechanism to strengthen shape feature extraction for elongated targets such as drill pipes, effectively improving detection accuracy in low-light scenarios, though with elevated missed detection rates in multi-target occlusion and high-noise coupling scenarios. Other studies have optimized feature fusion modules to improve multi-scale feature interaction efficiency. Li et al. [20] introduced the ECA attention mechanism and weighted CIoU loss function based on YOLOv11, enhancing detection robustness under low-light environments, but without fully adapting to the dynamic feature variations in multi-scale targets in underground mines. Most existing studies focus on addressing single or partial interference factors, lacking systematic adaptation to the multi-factor coupled interference of “low light, high noise, multi-scale, and strong occlusion” in underground coal mine environments, making it difficult to achieve coordinated optimization of detection accuracy, robustness, and lightweight design.

The main contributions of this work are summarized below, with each module systematically addressing a core bottleneck in existing detectors in underground coal mine scenarios.

First, we design a dynamic multi-scale global perception enhancement module (DMGPEM) to replace the static C3k2 modules in the YOLOv11n backbone. This module adopts dynamic depth-wise convolution and a residual enhancement mechanism, enabling adaptive capture of multi-scale target features and enhanced feature representation under low-light conditions.

Furthermore, we propose a lightweight cross-attention (LCA) module integrated into the neck network. Built on a dual-branch cross-guidance mechanism, the module suppresses dust-induced noise while enabling efficient fusion of shallow detail features and deep semantic features, with minimal computational overhead via depth-wise separable convolution.

Finally, we introduce a Reparameterized stem (RepStem) module to replace the original single-branch stem of YOLOv11n. The module uses multi-branch parallel downsampling to reduce critical information loss in the initial feature extraction stage, and achieves zero additional inference cost via structural reparameterization, suitable for edge deployment in underground mines.

Among these, DMGPEM is a novel customized module designed for underground coal mine scenarios, and LCA and RepStem are adapted improvements based on existing classic structures, both of which are tailored to solve the unique detection challenges in underground coal mines. The three proposed modules are designed to address three core challenges in underground mine detection: adaptive multi-scale feature extraction, noise-robust feature fusion, and minimal information loss during downsampling. Their synergistic effect is validated via ablation studies and comparative experiments. This research provides a technical reference for object detection in underground coal mine scenarios and has potential engineering application value for intelligent mine safety monitoring.

## 2. Methods and Principles

This section first introduces the baseline YOLOv11n architecture, then details the overall structure and three core optimized modules of the proposed DLR-YOLO model.

### 2.1. YOLOv11n Architecture

**Figure 1:**
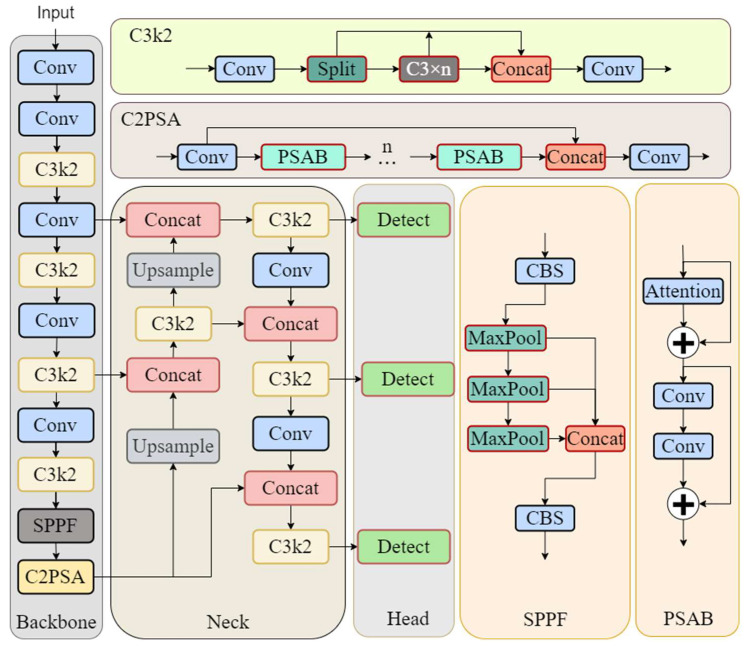
Architecture of the YOLOv11 network.

You only look once (YOLO), as a classic model in object detection, has undergone multiple iterations of optimization since its release in 2016 [21], gradually becoming a preferred solution for deployment in complex industrial scenarios. YOLOv11, while inheriting the detection accuracy advantages of its predecessors, focuses on upgrading computational efficiency and multi-task adaptability, demonstrating stronger practical value in scenarios such as low-computing-power device deployment and complex environment object detection. The model continues the classic backbone–neck–head three-module architecture in object detection, with targeted innovative designs incorporated into each core component to achieve coordinated improvement in performance and efficiency (see Figure 1):

(1) Backbone Network: The C3k2 module reconstructs feature extraction through repeated convolution and efficient information flow aggregation. Feature reuse strengthens deep feature representation while reducing redundant computation. The C2PSA attention module employs grouped interaction and parallel attention mechanisms to filter background redundancy and enhance target feature perception with controllable computational overhead.
(2) Neck Network: The PAFPN structure enables multi-scale feature fusion by introducing PANet’s bottom-up aggregation path alongside the traditional top-down path. This bidirectional mechanism achieves precise fusion of deep semantic and shallow detail features, effectively alleviating multi-scale target feature fragmentation and outputting three-scale feature branches.
(3) Head Network: The decoupled design separates classification and regression into independent branches, avoiding feature interference. Depth-wise separable convolution replaces standard convolution, reducing parameters and computation while maintaining accuracy, suitable for edge device deployment.

Evaluation results on the COCO dataset demonstrate that YOLOv11 not only excels in core accuracy metrics such as mAP@50 but also maintains lightweight structural characteristics in scenarios with complex backgrounds and dense small targets. Based on differences in network depth and channel count, the YOLOv11 series is divided into five model variants: YOLOv11n, YOLOv11s, YOLOv11m, YOLOv11L, and YOLOv11x, among which YOLOv11n features “minimum parameters and fastest inference speed” as its core advantages, matching the stringent requirements of edge device deployment scenarios such as comprehensive mining faces in underground coal mines. Therefore, this paper selects YOLOv11n as the baseline model for personnel and equipment detection research in complex underground coal mine environments.

### 2.2. DLR-YOLO Model Architecture

**Figure 2:**
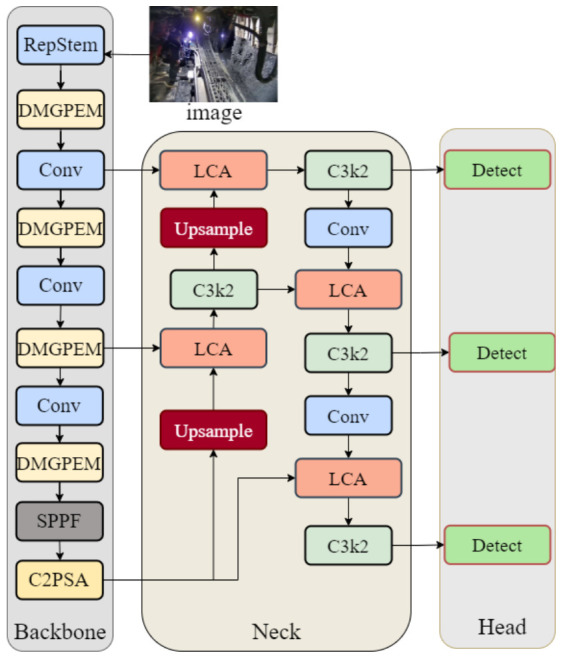
Architecture of DLR-YOLO network.

Based on the YOLOv11n architecture, this study proposes a novel object detector, DLR-YOLO, to improve detection performance in complex underground coal mine environments via targeted structural optimization. DLR-YOLO includes three core improvements, with its overall architecture shown in Figure 2. The architecture is designed to optimize three critical stages of the detection pipeline: initial feature extraction, deep feature enhancement, and multi-scale feature fusion, forming a complete end-to-end optimized detector.

Figure 2 clearly illustrates the hierarchical structure and module deployment positions of DLR-YOLO: input images first undergo initial feature extraction and downsampling through the reparameterized Repstem module, replacing the original YOLOv11n’s single-branch Stem to reduce critical information loss; in the backbone network, traditional C3k2 modules are replaced by DMGPEM, strengthening multi-scale target feature capture and low-light environment adaptation through dynamic convolution and residual enhancement mechanisms; the neck network integrates an LCA module on the basis of the PAFPN structure, replacing traditional feature concatenation operations to achieve efficient deep–shallow feature fusion and dust noise suppression; finally, classification and regression tasks are completed through decoupled branches in the head network, outputting detection results.

The three modules follow a progressive complementary functional logic with no functional redundancy: the RepStem module first preserves the fine-grained features of small targets in the initial downsampling stage to reduce information loss; the DMGPEM then adaptively extracts multi-scale features of targets under drastic scale changes and low illumination; and finally, the LCA module suppresses dust-induced background noise while fusing shallow detail and deep semantic features. The three modules form a complete “feature preservation–adaptive extraction–noise suppression and fusion” pipeline.

### 2.3. DMGPEM

**Figure 3:**
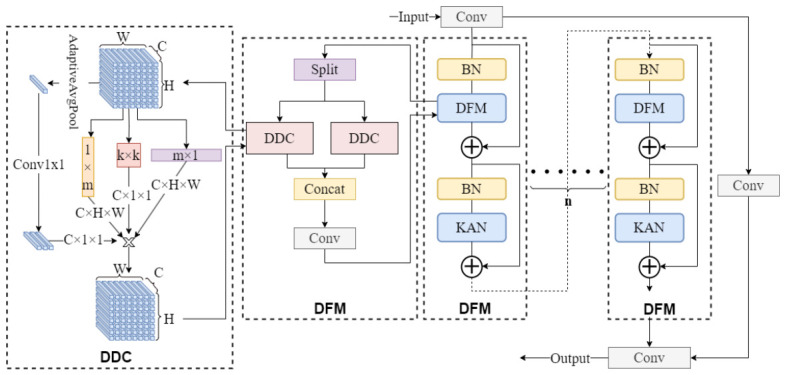
Structure of dynamic multi-scale global perception enhancement module.

Traditional convolutional neural networks possess fixed static characteristics in their convolution kernels, presenting significant limitations when processing underground coal mine scene inputs with complex multi-scale features and varying target morphologies, and are unable to precisely adapt to dynamic changes in target features. To address this, we design the DMGPEM, constructing a flexible and efficient convolutional architecture to break through this bottleneck and deploying it in the model backbone network to replace traditional C3k2 modules. The detailed structure of the proposed DMGPEM is illustrated in Figure 3. Compared with the static convolution in the C3k2 module, the dynamic multi-scale design of DMGPEM is improved to adapt to the drastic scale variations and low-light weak features of targets in underground coal mine scenarios.

The core architecture of DMGPEM consists of multiple stacked residual enhanced units (REU). Each REU achieves cross-layer information fusion through residual addition, where convolution-processed feature maps are added to the original inputs. This mechanism ensures efficient transmission of both low-level and high-level features while alleviating gradient vanishing in deep network training.

Each REU embeds a dynamic feature mixer (DFM) unit as its core component. The DFM constructs a multi-scale feature extraction paradigm by combining multiple dynamic depth-wise convolution (DDC) instances with different kernel sizes. Input channels are divided into groups, processed in parallel by independent DDC modules, and aggregated through 1 × 1 convolution. This design enables precise capture of target features at different scales, significantly enhancing adaptability to target diversity in underground coal mine scenarios.

The core advantage of DMGPEM stems from the dynamic depth-wise convolution mechanism, which assigns adaptive weights to each convolution kernel. This enables dynamic adjustment during forward propagation based on input characteristics such as weak features in low-light regions and multi-scale target superposition [22]. Specifically, global context is first extracted by compressing the spatial dimensions to 1 × 1, after which weight vectors are generated for the three convolutional branches and normalized to form a stable probability distribution for reliable feature fusion.

During forward propagation, after input feature maps are processed in parallel through three convolution paths, outputs from each path are weighted and summed according to their corresponding weights to generate aggregated feature maps; these feature maps then undergo batch normalization (BN) and Kolmogorov–Arnold network (KAN) activation function processing, finally outputting optimized feature results. KAN is a novel neural network architecture derived from the Kolmogorov–Arnold representation theorem [23], with its core innovation being the abandonment of traditional neural networks’ fixed nonlinear activation functions—each connecting edge corresponds to a learnable univariate function, with input signals aggregated and summed at nodes.

(1) $$KAN\left(x\right)=\sum_{j=1}^{n_{out}}\phi_{j}\left(\cdot \right)\left(\sigma \sum_{i=1}^{n_{in}}\phi_{i,j}\left(x_{i}\right)\right),$$

where x denotes the input vector; $n_{\mathrm{in}}$ represents the input feature dimension; $n_{out}$ represents the output feature dimension; $\phi_{i,j}\;$ is the learnable function between the i input node and the j output node; $\phi_{j}\left(\cdot \right)$ is the aggregation function at the output node; and σ is the activation function.

### 2.4. LCA Module

Underground coal mine environments pose substantial challenges to feature fusion because targets often exhibit weak contrast against the background under low illumination, leading to insufficient feature responses and ambiguous boundaries. In addition, high dust concentrations introduce strong noise redundancy, and conventional fusion operations tend to propagate or even amplify these artifacts during multi-scale aggregation. Meanwhile, objects of interest frequently appear at multiple scales, and the resulting mismatch between shallow detail features and deep semantic features can disrupt cross-scale alignment, thereby limiting the effectiveness of attention mechanisms that rely on a single branch or a single level of representation.

**Figure 4:**
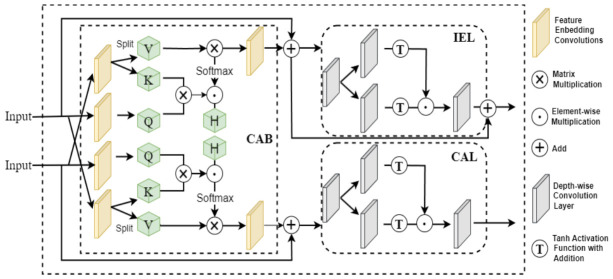
Structure of lightweight cross-attention module.

The traditional YOLOv11n neck relies on channel concatenation or self-attention mechanisms. The former lacks feature interaction capability; the latter’s quadratic computational complexity limits edge device deployment. The LCA module [24], originating from low-light image enhancement, achieves coordinated optimization of brightness and target features with low computational overhead. This paper introduces LCA into the DLR-YOLO neck layer to enable efficient deep-shallow feature fusion while suppressing dust noise (see Figure 4).

The LCA module follows four stages: input adaptation, cross interaction, coordinated enhancement, and residual fusion. An adaptive unit using Conv1×1 convolution and identity mapping addresses channel dimension differences between shallow and deep features. Conv1×1 maps mismatched dimensions while identity mapping preserves matched features, solving channel compatibility with minimal computational overhead.

To alleviate feature distribution fluctuations caused by underground mine image noise, before features enter the cross-interaction stage, channel-priority layer normalization operations are introduced to standardize both dual-branch features after adaptation:

(2) $$\hat{X}=\gamma \times \frac{X-E\left[X\right]}{\sqrt{Var\left[X\right]+\varepsilon }}+\beta ,$$

where $E\left[X\right]$ and $Var\left[X\right]$ are the mean and variance of features in the channel dimension, γ and β are learnable parameters, and $\varepsilon =10^{-6}$ is a regularization term to prevent division by zero. This operation stabilizes feature distribution, alleviating feature fluctuations caused by underground mine image noise, providing more robust input for subsequent cross-attention computation.

The cross-attention block (CAB), as the core interaction unit of LCA, has the design goal of achieving bidirectional information transmission between “source features–guidance features,” letting brightness-related features guide target feature denoising while target features constrain the rationality of brightness enhancement. The module projects source features into query vectors through 1 × 1 convolution, projects guidance features into key and value vectors, and expands the receptive field through depth-wise separable convolution to capture local texture information of underground mine targets. Subsequently, Q, K, and V are split by head count to construct multi-head cross-attention, enhancing the diversity of feature interaction and adapting to multi-scale target feature capture. A learnable temperature parameter is introduced to adjust attention distribution, avoiding excessive interference from single bright regions:

(3) $$Attn_{h}=\text{Softmax}\left(\frac{Q_{h}K_{h}^{⊤}}{\sqrt{d_{k}}\times \tau_{h}}\right),$$

(4) $$O_{h}=Attn_{h}\times V_{h},$$

where $Q_{h}$, $K_{h}$ and $V_{h}$ denote the query, key, and value matrices projected from source and guidance features, respectively; $d_{k}$ is the dimensionality of each attention head; and $\tau_{h}$ is a learnable temperature parameter that controls the sharpness of the attention distribution.

After concatenating multi-head attention outputs, projection back to the target channel count is completed through 1 × 1 convolution, completing the cross-interaction feature output. Subsequently, based on the Retinex theory, IEL decomposes cross-interacted features into illumination and reflectance components, solving the problem of weak low-light feature response in underground coal mines through nonlinear enhancement. First, feature decomposition is performed, decomposing features into two parallel branches (illumination) and (reflectance) through 1 × 1 convolution. Then, activation functions are used to enhance weak light region feature response while avoiding oversaturation in bright regions. The enhancement process is formulated as

(5) $${X_{L}}^{\prime }=\text{tanh}\left(\text{DWConv}\left(X_{L}\right)\right)+X_{L};$$

(6) $${X_{R}}^{\prime }=\text{tanh}\left(\text{DWConv}\left(X_{R}\right)\right)+X_{R}.$$

To avoid information loss during feature enhancement, residual connections are used to fuse IEL output with source features after input adaptation, finally obtaining the output features of the LCA module:

(7) $$O_{IEL}=\text{Conv}1\times 1\left(X_{L}\prime ⊙X_{R}\prime \right),$$

(8) $$O_{LCA}\;=X_{\mathrm{adapt}}\;+\;O_{IEL}$$

The LCA module is designed for three key requirements of underground coal mine detection: first, compared with traditional self-attention and simple feature concatenation operations, the dual-branch cross-guidance design of LCA is adapted from low-light image enhancement methods to adapt to complex underground environments; second, the module maintains low computational and parameter overhead via depth-wise separable convolution and 1 × 1 convolution projection, compared with traditional self-attention mechanisms; third, it achieves efficient bidirectional fusion of shallow detail features and deep semantic features and alleviates the challenge of incomplete feature representation across multi-scale targets.

### 2.5. RepStem Module

Underground coal mine scenarios characterized by small targets, low-resolution imagery, and complex backgrounds impose stringent requirements on initial feature extraction. The conventional YOLO stem stage employs a single-path 3 × 3 convolution with stride = 2 for simultaneous downsampling and channel expansion, which inherently limits the receptive field and causes critical information loss during the initial feature encoding phase. To address this limitation, this paper introduces the RepStem Module [25], originally proposed in FastViT for efficient hybrid vision transformers, to replace YOLOv11n’s single-branch stem architecture.

**Figure 5:**
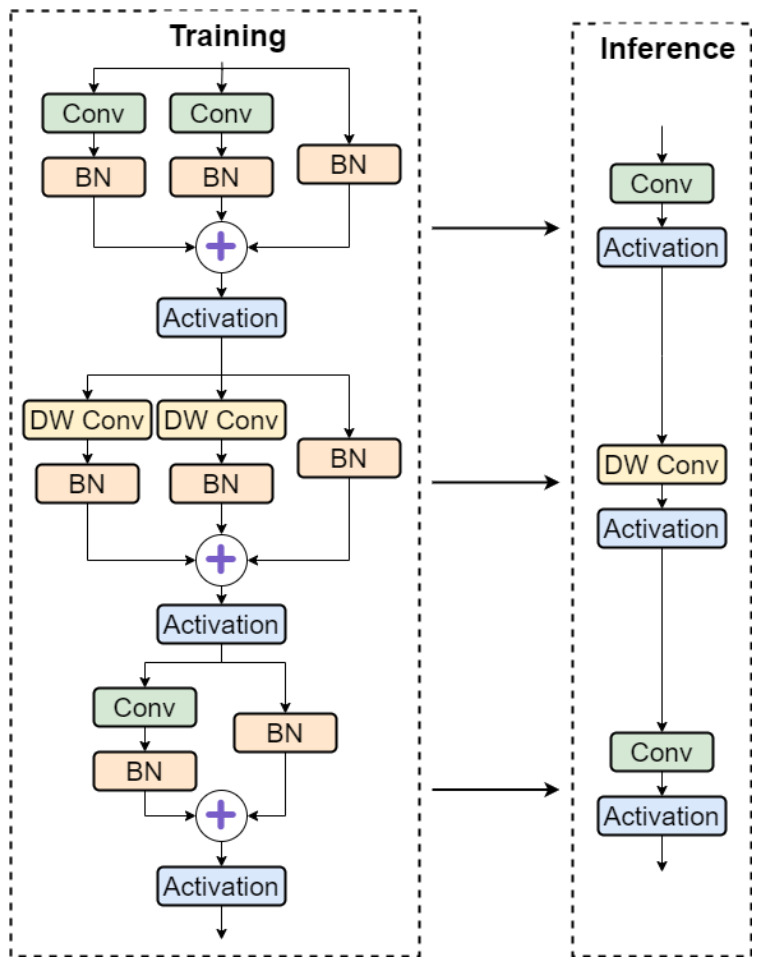
Structure of RepStem module.

As illustrated in Figure 5, RepStem adopts a hierarchical multi-branch parallel architecture comprising three sequential stages. In the first stage, two parallel standard convolution branches with different kernel sizes operate alongside a batch normalization (BN) identity branch, where the outputs are element-wise summed and passed through an activation function. The second stage employs dual depth-wise convolution (DW Conv) branches combined with a BN branch to capture spatial features with minimal parameter overhead. The third stage utilizes a standard convolution branch paired with a BN branch for final channel projection. This multi-branch topology enables comprehensive low-level feature extraction by simultaneously aggregating channel information through different receptive fields while preserving fine-grained spatial details essential for small target detection in underground environments.

The key innovation of RepStem lies in its structural reparameterization mechanism. During training, the multi-branch architecture provides enriched gradient flow paths that alleviate the gradient vanishing problem and enhance training stability. During inference, the parallel branches within each stage are mathematically equivalent to a single convolution operation through kernel fusion, where the weights from multiple branches are merged into unified convolutional kernels. This “multi-branch training, single-branch inference” paradigm ensures that the enhanced feature representation capability incurs no additional computational overhead during deployment, making RepStem particularly suitable for edge device deployment in coal mine monitoring systems where both detection accuracy and inference efficiency are critical requirements.

## 3. Experimental Results and Analysis

This section presents the experimental setup and results of the proposed DLR-YOLO model, including dataset description, experimental configuration, evaluation metrics, and systematic analysis of ablation experiments, comparative experiments, and qualitative detection results.

### 3.1. Dataset Description

**Figure 6:**
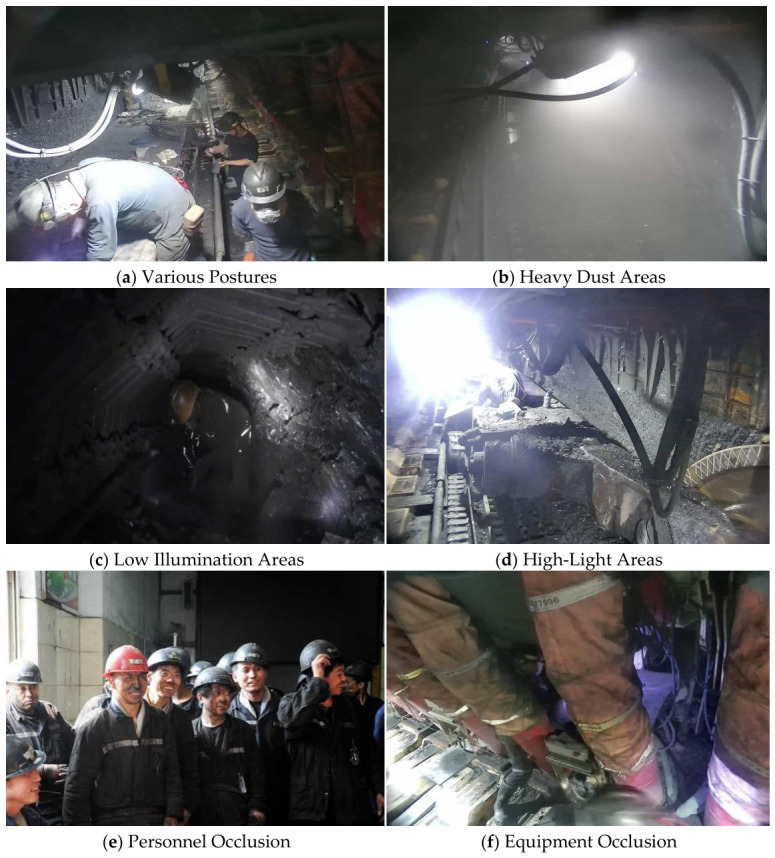
Typical scenario samples of the self-built underground coal mine dataset: (**a**) samples with various postures of miners, (**b**) samples in heavy dust areas, (**c**) samples in low illumination areas, (**d**) samples in high-light areas, (**e**) samples with personnel occlusion, and (**f**) samples with equipment occlusion.

To fully disclose the distribution of our self-built underground coal mine dataset and ensure the full reproducibility of our experiments, we detail the complete dataset partitioning and annotation specifications below. The dataset is constructed from real underground coal mine operation images collected from a coal mine in Henan Province, China, covering tunneling faces, transport tunnels, and equipment maintenance areas, with typical interference factors including low illumination, high dust, target occlusion, and drastic scale variations (Figure 6). All images are annotated using the open-source LabelImg tool (v1.8.6) in Pascal VOC format, with a dual-annotator cross-validation process (IoU ≥ 0.7 is set as the qualified annotation standard) to guarantee labeling quality. The dataset is split into training, validation, and test sets at a fixed ratio of 8:1:1, with no data leakage across splits. The detailed category distribution is shown in Table 1.

### 3.2. Experimental Setup

To address the fairness concerns of comparative experiments, all baseline models and our proposed DLR-YOLO were trained and evaluated under completely identical settings. All performance metrics of baseline models were obtained from our independent training under the unified configuration below, with no direct citation of third-party reported results. The Unified Core Experimental Control Variables are shown in Table 2.

### 3.3. Evaluation Metrics

The model’s performance evaluation metrics adopt Precision (P), Recall (R), Average precision (AP), Mean average precision (mAP), Computational cost (GFLOPs), parameter count, Frame rate (FPS), and F1-measure (F1) as evaluation indicators. P refers to the percentage of correctly predicted foreground targets among all targets predicted as foreground; R represents the percentage of correctly detected foreground targets among all foreground targets; AP is the area under the P-R curve corresponding to each category, and mAP is the average of P-R curve areas across all categories; FPS represents the number of images processed by the model per second; GFLOPs and Params are used to evaluate model computational complexity and lightweight level; and the F1-measure is used as a comprehensive indicator.

### 3.4. Experimental Results

This section systematically analyzes the experimental results from multiple dimensions, including a performance comparison with the baseline model, effectiveness analysis of each module, ablation study, comparison with state-of-the-art models, and qualitative detection result analysis.

#### 3.4.1. Detection Performance Comparison Before and After Model Improvement

**Figure 7:**
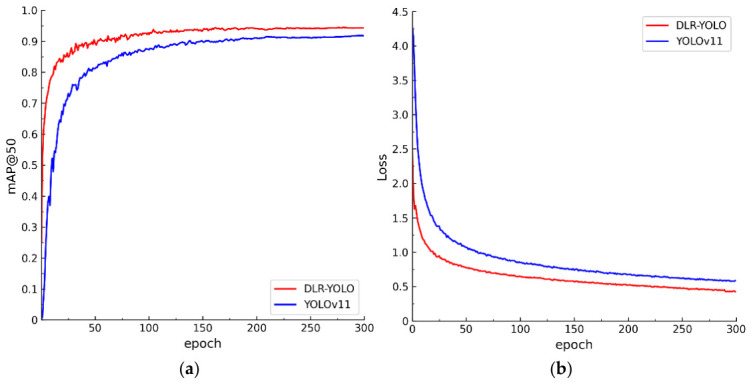
Comparison of training process curves between two types of models: (**a**) mAP@0.5 curve and (**b**) loss curve.

**Figure 8:**
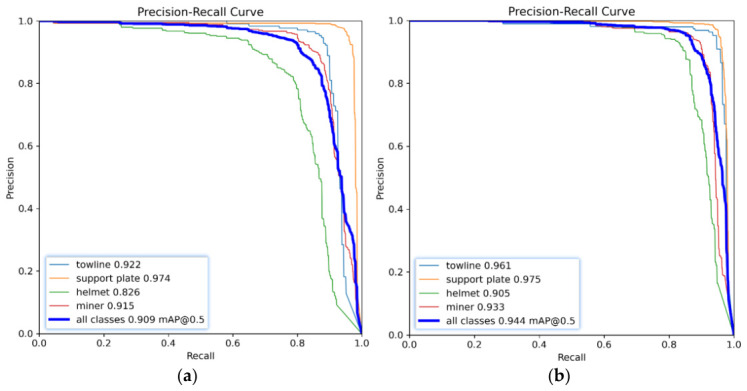
Comparison of the P-R curves of two types of models on the validation set: (**a**) P-R curve of YOLOv11n and (**b**) P-R curve of DLR-YOLO.

To intuitively compare the detection performance differences between DLR-YOLO and the original YOLOv11n model, this section conducts systematic analysis through training process curves and validation set P-R curves, with results shown in Figure 7 and Figure 8. Figure 7a presents the mAP@0.5 change trends of both models across 300 training epochs, showing that the original YOLOv11n model has slower convergence speed in early training and gradually enters a performance plateau after epoch 200, with final mAP@0.5 stabilizing at 90.9%; while DLR-YOLO, through the synergistic action of DMGPEM, LCA, and RepStem modules, not only converges significantly faster, approaching the original model’s final performance around 150 epochs, but continues to steadily improve, reaching a final mAP@0.5 of 94.4%, representing a 3.5 percentage point improvement over the original model, fully demonstrating the optimization of feature extraction and fusion efficiency by the improved structure. Figure 7b’s loss curves further validate this advantage: DLR-YOLO’s training loss decreases faster, with no obvious oscillation in late training stages, consistently maintaining lower loss levels than the original YOLOv11n, indicating that the improved modules effectively alleviate gradient vanishing and feature loss problems, improving model training stability and convergence quality.

In validation set P-R curve comparison (Figure 8), YOLOv11n’s P-R curve (Figure 8a) shows certain differences in AP across categories, with support plate AP being the highest (0.974) and helmet AP being relatively lower (0.826), and all categories’ mAP@0.5 at 90.9%, reflecting insufficient balance between detection precision and recall for some targets (such as helmets susceptible to occlusion and lighting effects) in low-light, high-dust underground coal mine environments under the original model. DLR-YOLO’s P-R curve (Figure 8b) shifts overall toward the upper right corner of the coordinates, with enclosed areas of P-R curves for all categories expanding to varying degrees, with helmet detection performance showing the most significant improvement, while AP values for miners, towlines, and support plates are also effectively optimized, with the final mAP@0.5 improving to 94.4%, proving that through improvements in dynamic multi-scale feature extraction, lightweight cross-attention fusion, and reparameterized initial feature representation, the model can better cope with low-light noise interference and large-scale variation in targets, fragmented feature representation, and frequent occlusion in complex underground coal mine environments, achieving a better balance between precision and recall and providing more reliable performance support for underground target detection.

To further evaluate the stability of the proposed model, we performed three independent repeated experiments under the same settings. The mAP@50 of DLR-YOLO remained stable at 94.4% ± 0.24%, with a maximum deviation of less than 0.3%, indicating that the model has reliable performance.

#### 3.4.2. DMGPEM Effectiveness Analysis

To validate the effectiveness of the DMGPEM, this paper designs comparative experiments embedding it in the backbone, neck, and combined positions in YOLOv11n, with evaluation compared to the baseline model, and results shown in Table 3. The baseline model’s P, R, mAP@50, and mAP@50–95 are 90.6%, 85.9%, 90.9%, 61.0% respectively; when embedded only in the backbone, all metrics improve most significantly, reaching 92.1%, 86.7%, 92.6%, 63.2% respectively; when embedded only in the neck, the metrics are 91.4%, 87.2%, 92.1%, 62.3%; when both are combined, the metrics are 91.7%, 86.8%, 92.2%, 62.5%. Experiments demonstrate that the DMGPEM can effectively improve target detection performance in underground coal mine environments, with optimal effectiveness when deployed at the backbone position, where its dynamic multi-scale perception and residual enhancement design can strengthen feature extraction capability, adapting to multi-scale target detection requirements in complex scenarios.

#### 3.4.3. LCA Effectiveness Analysis

To validate the effectiveness of the LCA module in object detection in underground coal mine environments, this paper compares it with mainstream neck feature fusion strategies including BiFPN [26], ASFF [27], SFAM [28], SSFF [29], and GDSAF [30], evaluating performance from four core metrics: mAP@50, parameter count, computational cost, and frame rate, with results shown in Table 4. Experimental results clearly show that the LCA strategy achieves an mAP@50 of 63.1%, only slightly lower than BiFPN’s 63.4%, at a leading level in detection accuracy; meanwhile, LCA’s parameter count is only 2.4 M, the lowest among all compared strategies, its computational cost maintains the favorable level of 6.3 GFLOPs, and its frame rate reaches 172.0 FPS, outperforming other compared strategies. This demonstrates that LCA achieves parameter minimization and inference efficiency maximization while ensuring detection accuracy, with its “dual-branch cross-guidance + lightweight structure” design effectively adapting to the computational constraints of underground mine edge devices, solving both the noise amplification and feature fragmentation problems in traditional feature fusion while balancing detection performance and deployment efficiency, serving as an efficient feature fusion solution for the neck part of object detection models in complex underground coal mine environments.

#### 3.4.4. RepStem Effectiveness Analysis

To validate the effectiveness of the RepStem module, this paper designs comparative experiments benchmarking it against mainstream downsampling improvement strategies including SPDConv [31], WaveletPool [32], LDConv [33], ADown, and SRFD [34], with evaluation based on four core metrics, P, R, mAP@50, mAP@50–95, and results shown in Table 5. Experimental data shows that compared to other strategies, RepStem’s detection performance metrics are all at leading levels: Precision P reaches 91.5%, Recall R is 87.0%, mAP@50 and mAP@50–95 improve to 92.4% and 63.4%, respectively, significantly higher than in traditional strategies like SPDConv (mAP@50 87.4%) and WaveletPool (mAP@50 88.9%), and superior to the currently better-performing SRFD (mAP@50 92.0%, mAP@50–95 62.5%). This demonstrates that RepStem, leveraging a multi-branch parallel and reparameterization design, both fully extracts low-level features from input images and reduces information loss during downsampling, while also balancing detection accuracy and deployment efficiency through “multi-branch for performance during training, single-branch for speed during inference” characteristics. Its strengthening design for initial feature representation capability can effectively adapt to detection requirements for small targets and low-resolution inputs in underground coal mine scenarios, validating the advantages and effectiveness of this module in improving initial feature extraction.

#### 3.4.5. Ablation Experiments

To validate the independent contributions and synergistic effects of the three core modules (DMGPEM, LCA, and RepStem), this paper designs systematic ablation experiments, with results shown in Table 6. Using the baseline model without any improvements as reference, embedding each module individually significantly improves detection performance: adding DMGPEM increases mAP@50 to 92.6% and mAP@50–95 to 63.7%; adding LCA achieves an mAP@50 of 92.9% with only 2.4M parameters and a 172.0 FPS frame rate, demonstrating outstanding lightweight advantages; and adding RepStem yields an mAP@50 of 92.4% and an mAP@50–95 of 63.4%, significantly enhancing initial feature extraction capability.

When combining modules pairwise, synergistic gains become evident: DMGPEM + LCA achieves an mAP@50 of 93.7%; DMGPEM + RepStem attains the highest Precision (94.1%); and LCA + RepStem reaches a Recall of 89.0% and mAP@50–95 of 65.9%. When all three modules are simultaneously embedded (i.e., DLR-YOLO), all performance metrics reach optimal levels, P 93.9%, R 89.7%, mAP@50 94.4%, mAP@50–95 66.7%, while maintaining 2.7 M parameters, 6.6 GFLOPs in computational cost, and a 157.1 FPS frame rate, without a substantial increase in complexity due to structural optimization.

These experiments demonstrate that the three modules form an effective synergy across feature extraction, feature fusion, and initial feature representation stages—each fulfilling its core function while complementing each other to enhance overall performance—validating the rationality and effectiveness of the DLR-YOLO model architecture design.

#### 3.4.6. Comparison with State-of-the-Art Models

To ensure an objective and comprehensive assessment, we compare DLR-YOLO with multiple categories of state-of-the-art methods: traditional two-stage detectors (Faster R-CNN), one-stage anchor-based detectors (RetinaNet [35], YOLOv5s [36], YOLOv8s [37]), the latest anchor-free DETR-based detector (RT-DETR [38]), and the most recent YOLO variants (third-party open-source YOLOv12 [39], YOLOv13 [40] implementations, which are not official stable releases from Ultralytics), with evaluation conducted on eight core metrics—P, R, F1-measure, mAP@50, mAP@50–95, parameter count, computational cost, and frame rate—and the results shown in Table 7. Experimental data clearly demonstrates that DLR-YOLO achieves competitive comprehensive performance in detection accuracy: Precision P reaches 93.9%, Recall R is 89.7%, F1-measure reaches 91.8%, and mAP@50 and mAP@50–95 reach 94.4% and 66.7%, respectively, not only substantially surpassing traditional models such as (Faster R-CNN mAP@50 69.1%, RetinaNet mAP@50 64.7%) and RT-DETR (mAP@50 81.2%), but also improving by over 3 percentage points compared to the latest YOLO series models (YOLOv12 mAP@50 91.5%, YOLOv13 mAP@50 91.0%). In its lightweight and inference efficiency aspects, DLR-YOLO’s parameter count (2.7M) and computational cost (6.6 GFLOPs) are comparable to YOLOv8s, v12, and v13, maintaining extremely lightweight characteristics, while its frame rate (157.1 FPS) is significantly higher than traditional models and RT-DETR and superior to YOLOv5s through YOLOv12 series models. This demonstrates that DLR-YOLO, through synergistic optimization of the DMGPEM, LCA, and RepStem modules, achieves dual breakthroughs in detection accuracy and inference efficiency without increasing model complexity, with overall performance superior to mainstream object detection models.

#### 3.4.7. Detection Results Analysis

**Figure 9:**
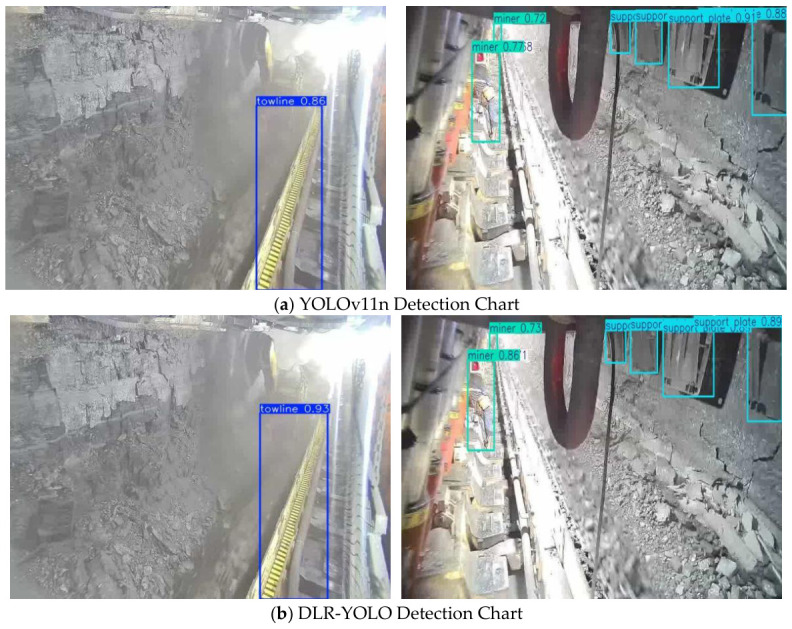
Qualitative comparison of detection results between YOLOv11n and the proposed DLR-YOLO: (**a**) detection result of YOLOv11n, and (**b**) detection result of DLR-YOLO.

We qualitatively compare the detection performance between YOLOv11n and DLR-YOLO in real underground coal mine scenarios. Experimental results shown in Figure 9 indicate that the original YOLOv11n has obvious shortcomings under low illumination, high dust, and target occlusion scenarios: detection confidence for towlines is only 0.86, detection confidence for miners is 0.72, with slight missed detection signs in equipment occlusion areas, and category recognition for support plates has ambiguity issues. In contrast, DLR-YOLO significantly optimizes detection performance for all target categories: towline detection confidence improves to 0.93, miner detection confidence stabilizes above 0.73, with no obvious missed or false detections in the tested typical extreme scenarios such as heavy dust and dense personnel occlusion, and category recognition accuracy substantially improves. These qualitative results strongly support that the proposed DMGPEM, LCA, and RepStem modules effectively mitigate the core challenges of weak target feature representation and severe noise interference in underground coal mine scenarios, leading to improved detection confidence and target recognition completeness.

**Figure 10:**
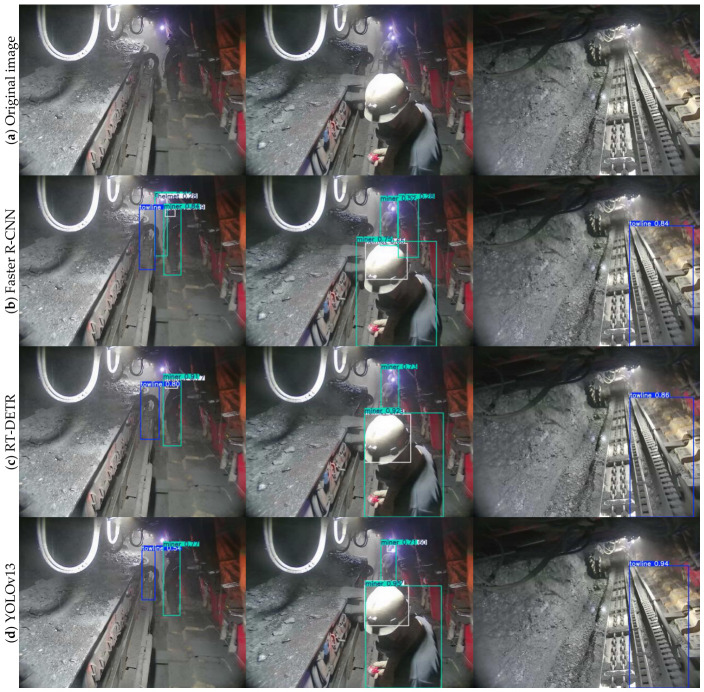

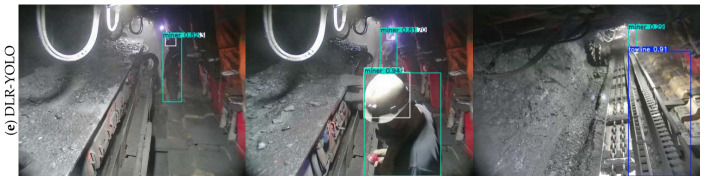
Qualitative comparison of detection bounding box performance among mainstream models: (**a**) original underground coal mine image, (**b**) detection result of Faster R-CNN, (**c**) detection result of RT-DETR, (**d**) detection result of YOLOv13, and (**e**) detection result of the proposed DLR-YOLO.

To further verify the comprehensive superiority of the proposed DLR-YOLO over mainstream state-of-the-art object detectors in complex underground coal mine scenarios, we conduct a multi-model qualitative comparison of detection box localization and recognition effects, with the results presented in Figure 10. This figure compares the detection performance of four representative mainstream detectors (the two-stage Faster R-CNN, the anchor-free RT-DETR, and the latest YOLO variant YOLOv13) and the proposed DLR-YOLO on the same set of underground coal mine images, covering typical challenging scenarios including low illumination, dense target occlusion, high dust interference, and low-contrast weak feature targets. The comparative results show that Faster R-CNN and RT-DETR have obvious missed detection problems for occluded targets and small-scale targets, with generally low detection confidence and large deviations in the bounding box positioning accuracy; YOLOv13 exhibits partial false detections and missed detections in small target and low-light scenarios. In contrast, the proposed DLR-YOLO achieves accurate and complete detection for all four target categories in all tested challenging scenarios, with higher and more stable detection confidence, more precise bounding box positioning that fits the target contour better, no obvious missed or false detections, and maintains excellent detection robustness even under the coupled interference of low illumination, high dust, and target occlusion. These qualitative visualization results are highly consistent with the quantitative performance improvement in the previous comparative experiments, further demonstrating that the proposed DLR-YOLO has better comprehensive detection performance and environmental adaptability.

**Figure 11:**
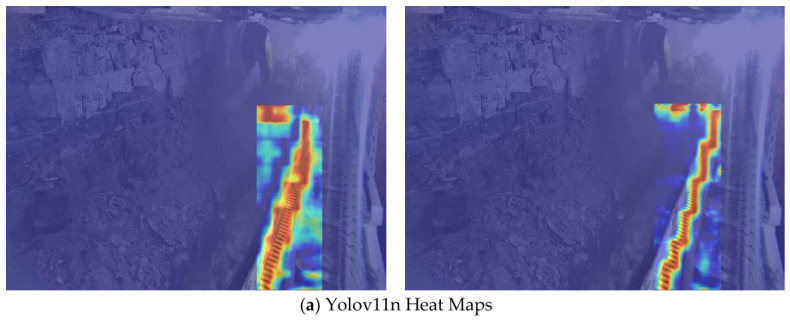

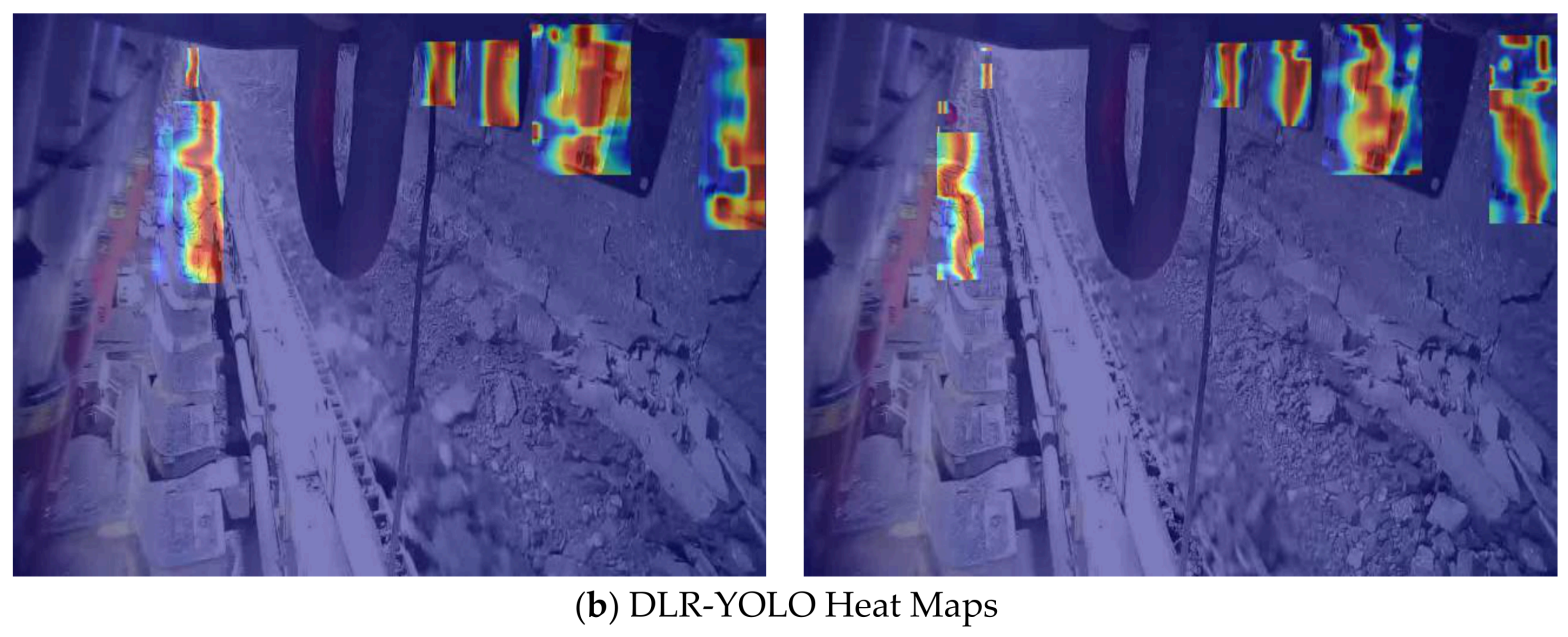
Comparison of attention heat maps between the baseline and the proposed model: (**a**) attention heat map of YOLOv11n, and (**b**) attention heat map of DLR-YOLO.

Further comparative analysis of the attention allocation mechanism differences through heat maps can more deeply reveal DLR-YOLO’s performance advantages. Quantitative analysis of the attention heatmap shows that the attention weight ratio of DLR-YOLO in the target region reaches 89.3%, which is 6.7% higher than the YOLOv11n baseline, and the invalid attention in the background area is significantly reduced. As shown in Figure 11a, YOLOv11n’s heat map shows scattered responses in background areas such as tunnel walls and dust interference regions, with insufficient attention focus on target edges, causing effective features to be diluted by irrelevant information; while DLR-YOLO’s heat map (Figure 11b) precisely concentrates on core target areas including miners, helmets, and towlines, with significantly reduced background response proportion and more complete attention coverage of target contours and details. Heat map visualization illustrates that the proposed modules guide the model to focus on core target regions (including miners, helmets, and towlines) while significantly suppressing responses to background interference such as tunnel walls and dust scattering. This attention behavior aligns with the quantitative improvements in detection accuracy, confidence, and robustness to environmental interference observed in our experiments.

## 4. Conclusions

This paper proposes DLR-YOLO, an improved object detector based on YOLOv11n, targeting the complex scenarios of low illumination, high dust concentration, large-scale variation in targets, fragmented feature representation, and frequent occlusion in underground coal mines. Through synergistic optimization of three core modules, it effectively mitigates key detection bottlenecks of the baseline YOLOv11n model in underground mine scenarios: the DMGPEM adapts to multi-scale target feature extraction; the LCA module achieves coordinated optimization of brightness and target features with low computational overhead while suppressing dust noise; the RepStem module ensures initial feature integrity and lightweight deployment efficiency.

Experimental results demonstrate that DLR-YOLO achieves 93.9% precision, 89.7% recall, 94.4% mAP@50, and 66.7% mAP@50–95 on the self-constructed underground coal mine dataset, improving its mAP@50 by 3.5 percentage points compared to the original YOLOv11n, while achieving a favorable trade-off between detection accuracy, model size, and inference speed with 2.7 M parameters, 6.6 GFLOPs in computational cost, and a 157.1 FPS frame rate. Its overall performance is superior to several representative recent detectors on our in-house dataset. The visualization results further validate the model’s high detection confidence and robustness to noise, dust, occlusion, and uneven illumination in complex scenarios.

However, this paper still has certain limitations: all experiments were conducted on a self-built dataset from a single coal mine, and the model was not verified on public object detection datasets. In addition, the self-constructed dataset contains only four basic target categories including miners and helmets, without covering key safety warning categories such as mechanical fault components and prohibited tools underground; generalization testing for different geological conditions such as thin coal seams and high-gas tunnels has not been conducted, and the model’s adaptability in diverse mine scenarios requires verification.

Future work will focus on concrete verification and targeted optimization corresponding to the current model’s limitations; we will expand the dataset to include more than eight categories with quantitative validation on sample distribution and detection stability by supplementing key safety targets such as mechanical fault components and prohibited tools, carry out cross-scene generalization verification on public coal mine datasets and data from mines with different geological conditions (thin coal seams, high-gas roadways) to test the model’s adaptability in diverse mine scenarios, integrate infrared-visible multi-modal fusion with contrast verification to enhance robustness in extreme low-light underground environments, and implement model quantization, pruning and embedded adaptation with deployment verification on mainstream mining edge devices to further improve the engineering practicality of the model.

## Figures and Tables

**Table 1:** Detailed distribution of the self-built underground coal mine dataset.

Target Category	Training Set	Validation Set	Test Set	Total Annotated Bounding Boxes	Class Proportion
miner	3187	399	402	3998	24.1%
helmet	2589	324	321	3234	19.5%
towline	1342	167	165	1674	10.1%
support plate	4988	624	627	6239	37.7%
Total	12,106	1514	1515	15,135	100%

**Table 2:** Unified core experimental control variables.

Category	Core Configuration Item	Unified Parameter for All Models
Dataset & Input Control	Input Image Resolution	640 × 640 pixels
	Dataset Partition	Fixed 8:1:1 train/validation/test split on our in-house underground coal mine dataset
	Data Augmentation	Mosaic (probability = 1.0) + horizontal flip (probability = 0.5); all model-specific built-in augmentations disabled
Training Pipeline Control	Total Training Epochs	300 epochs
	Early Stopping Criterion	training stops if validation mAP@50 does not improve for 20 consecutive epochs.
	Pre-trained Weights	Official COCO pre-trained weights from each model’s original authors
	Batch Size	8 (training), 1 (inference)
Optimization Hyperparameter Control	Optimizer	SGD (momentum = 0.9, weight decay = 0.0005)
	Learning Rate Schedule	Initial lr = 0.01, final lr coefficient = 0.01, unified step decay
Inference & Evaluation Control	Hardware & Software Environment	Windows 11 OS, NVIDIA GeForce RTX 5060 8GB GPU, AMD Ryzen 7 4800 CPU, 16 GB RAM; CUDA 12.9, PyTorch 2.2.1, Python 3.10
	FPS Calculation Rule	Average inference speed over 1000 test images after 100 warm-up iterations
	Evaluation Metrics	Standard COCO-format Precision, Recall, mAP@50, mAP@50–95

**Table 3:** Effectiveness analysis of DMGPEM.

Model	P/%	R/%	mAP@50/%	mAP@50–95/%
Baseline	90.6	85.9	90.9	61.0
+DMGPEM (Backbone)	92.1	86.7	92.6	63.2
+DMGPEM (Neck)	91.4	87.2	92.1	62.3
+DMGPEM (Neck, Backbone)	91.7	86.8	92.2	62.5

**Table 4:** Effectiveness analysis of LCA module.

Strategy	mAP@50/%	Params/M	GFLOPs	FPS
BiFPN	63.4	2.7	6.6	155.2
ASFF	60.5	2.6	6.7	158.3
SFAM	62.7	2.5	6.6	162.4
SSFF	59.8	2.6	6.5	163.1
GDSAF	61.4	2.5	6.3	168.7
LCA	63.1	2.4	6.3	172.0

**Table 5:** Effectiveness analysis of RepStem module.

Strategy	P/%	R/%	mAP@50/%	mAP@50–95/%
SPDConv	89.4	85.0	87.4	58.2
WaveletPool	89.7	86.1	88.9	59.3
LDConv	90.5	86.3	90.8	60.1
ADown	90.2	86.7	90.6	60.7
SRFD	90.8	87.2	92.0	62.5
RepStem	91.5	87.0	92.4	63.4

**Table 6:** Ablation study results. A check mark (√) indicates that the corresponding module is included in the model configuration.

DMGPEM	LCA	RepStem	P/%	R/%	mAP@50/%	mAP@50–95/%	Params/M	GFLOPs	FPS
			90.6	85.9	90.9	61.0	2.7	6.5	156.9
√			92.1	86.7	92.6	63.7	2.8	6.7	146.4
	√		92.3	86.7	92.9	63.1	2.4	6.3	172.0
		√	91.5	87.0	92.4	63.4	2.7	6.6	150.3
√	√		93.8	88.6	93.7	65.2	2.6	6.5	161.5
√		√	94.1	88.2	93.5	64.8	2.8	6.6	153.7
	√	√	93.2	89.0	93.7	65.9	2.5	6.5	164.9
√	√	√	93.9	89.7	94.4	66.7	2.7	6.6	157.1

**Table 7:** Performance comparison of DLR-YOLO compared to mainstream models.

Strategy	P/%	R/%	mAP@50/%	mAP@50–95/%	Params/M	GFLOPs	FPS	F1/%
Faster R-CNN	74.2	67.2	69.1	49.7	52.1	137.4	7.7	70.5
RetinaNet	71.0	63.5	64.7	44.5	48.5	126.7	10.3	67.0
RT-DETR	85.7	82.0	81.2	53.1	14.9	33.0	38.2	83.8
YOLOv5s	90.1	85.5	89.0	60.1	2.8	6.9	141.1	87.7
YOLOv8s	90.5	85.8	90.2	60.7	2.7	6.8	147.0	88.1
YOLOv12	91.0	86.3	91.5	62.3	2.7	6.6	150.7	88.6
YOLOv13	90.8	85.9	91.0	61.7	2.7	6.5	156.0	88.3
DLR-YOLO	93.9	89.7	94.4	66.7	2.7	6.6	157.1	91.8

## Data Availability

The original contributions presented in this study are included in the article. Further inquiries can be directed to the corresponding author.
